# Regional catastrophic health expenditure and health inequality in China

**DOI:** 10.3389/fpubh.2023.1193945

**Published:** 2023-10-12

**Authors:** Xinyue Wang, Yan Guo, Yang Qin, Stephen Nicholas, Elizabeth Maitland, Cai Liu

**Affiliations:** ^1^School of Management, Tianjin University of Traditional Chinese Medicine, Tianjin, China; ^2^Dispatching and Operation Department, Construction and Management Bureau of the North Hu Bei Water Transfer Project, Wuhan, China; ^3^Australian National Institute of Management and Commerce, Sydney, NSW, Australia; ^4^School of Economics and School of Management, Tianjin Normal University, Tianjin, China; ^5^Research Institute for International Strategies, Guangdong University of Foreign Studies, Guangzhou, China; ^6^Newcastle Business School, University of Newcastle, University Drive, Newcastle, NSW, Australia; ^7^School of Management, University of Liverpool, Liverpool, United Kingdom

**Keywords:** catastrophic health expenditures, regional differences, health inequality, China, random effect model, unbalanced panel dataset

## Abstract

**Background:**

Catastrophic health expenditures (CHE) can trigger illness-caused poverty and compound poverty-caused illness. Our study is the first regional comparative study to analyze CHE trends and health inequality in eastern, central and western China, exploring the differences and disparities across regions to make targeted health policy recommendations.

**Methods:**

Using data from China's Household Panel Study (CFPS), we selected Shanghai, Henan and Gansu as representative eastern-central-western regional provinces to construct a unique 5-year CHE unbalanced panel dataset. CHE incidence was measured by calculating headcount; CHE intensity was measured by overshoot and CHE inequality was estimated by concentration curves (CC) and the concentration index (CI). A random effect model was employed to analyze the impact of household head socio-economic characteristics, the household socio-economic characteristics and household health utilization on CHE incidence across the three regions.

**Results:**

The study found that the incidence and intensity of CHE decreased, but the degree of CHE inequality increased, across all three regions. For all regions, the trend of inequality first decreased and then increased. We also revealed significant differences across the eastern, central and western regions of China in CHE incidence, intensity, inequality and regional differences in the CHE influencing factors. Affected by factors such as the gap between the rich and the poor and the uneven distribution of medical resources, families in the eastern region who were unmarried, use supplementary medical insurance, and had members receiving outpatient treatment were more likely to experience CHE. Families with chronic diseases in the central and western regions were more likely to suffer CHE, and rural families in the western region were more likely to experience CHE.

**Conclusions:**

The trends and causes of CHE varied across the different regions, which requires a further tilt of medical resources to the central and western regions; improved prevention and financial support for chronic diseases households; and reform of the insurance reimbursement policy of outpatient medical insurance. On a regional basis, health policy should not only address CHE incidence and intensity, but also its inequality.

## Introduction

Universal health coverage (UHC) means that all people have access to the full range of quality health services they need, when and where they need them, without financial hardship. The goal to achieve UHC aligns with the 2030 Sustainable Development Goals (SDGs) set in 2015 and is also reflected in the objectives of China's health insurance schemes. All schemes require contributions from the insured and mixed financing from the local and central governments, with different benefit levels. NRCMS (New Rural Cooperative Medical Scheme) covers rural residents; URBMI (Urban Resident Basic Medical Insurance) covers the urban unemployed, retired, older adult, students, and children; UEBMI (Urban Employee Basic Medical Insurance) covers the formal employed urban residents and their families; and other medical insurance (free medical care and supplementary medical insurance) are related to employment status with varying benefits (1, 2).

Despite varying contribution levels and support from local government, patients still face significant out-of-pocket medical expenses, resulting in significant healthcare costs for sick people.

Catastrophic health expenditures can trigger illness-caused poverty and compound poverty-caused illness, forcing households to reduce non-medical consumption, worsen family quality of life, and plunge households into long-term debt and medical poverty (3). Catastrophic health expenditures (CHE) are out-of-pocket payments (OOP) for medical expenses not covered by health insurance, which are ≥40% of total household expenditure minus food spending (4–12). According to a CHE study of 59 countries, China suffered a CHE incidence of 13%, compared to 0.01% in the Czech Republic and Slovakia and 10.5% in Vietnam (13). However, CHE was not uniform across China, varying due to financial payments, treatment packages, and reimbursement levels across different health insurance policies, different demographic structures, and the diverse treatment of rural migrants. Xu identified a strong pro-poor inequity of CHE in the rural areas of Shanxi, a central region province (14), and Yang observed that empty-nest households were at higher CHE risk than non-empty-nest households in Shandong, an eastern province (15). In Jiangsu, also an eastern province, Li et al. (16) found that while critical illness insurance decreased the incidence of CHE, it increased the financial intensity of such events.

To address China's inter-regional inequality, the government has adopted targeted poverty alleviation strategies, especially for China's poorer central and western provinces (17). The health poverty alleviation project implemented a “triple guarantee,” comprising basic medical insurance, serious illness insurance, and medical assistance health insurance, to help households forced into illness-caused poverty and to act as a safety net for poverty-caused illness households (18).

Especially notable among policy successes is the national health insurance that protects households from poverty-related CHE. Covering more than 1.35 billion people, China's national basic medical insurance now reaches 95% of the population, with the number of people forced into poverty due to illness falling significantly from 28.5 million in 2014 to 969,000 in 2019 (19). On the supply side, significant investments have improved county-level medical facilities, and the number of qualified GPs has increased from 150,000 in 2013 to 410,000 in 2020 to achieve the national health reform target of 2.9 doctors per 10,000 residents (20).

However, significant regional differences persist in CHE, affecting both families and health facilities. Rather than regional studies, China CHE studies have mainly focused on the national level (21, 22) rural-urban comparisons (23) or single provinces. Only one study took an inter-provincial comparative perspective, finding the average rate of CHE in eastern Zhejiang province was 9.6% vs. 30.5% in western Qinghai province (24).

We undertake a regional comparative study of CHE. Our study recognizes that China experiences huge regional disparities in economic development level, health resource allocation, and population health status. For example, per capita GDP was RMB42000 in the eastern provinces but almost 50% lower in the central (RMB25960) and western (RMB24000) provinces (25). According to the 2021 China Health 2022 Statistical Yearbook (26), there were 394,513 health facilities in the east, but 323,989 in the central and 312,433 in the western provinces, and 6.1 million health personnel in the eastern region, but only 4 million in the central and western regions. The regional disparities were also reflected in health spending: RMB2.71 trillion in the eastern region compared to RMB1.27 trillion in the central region and RMB1.18 trillion in the western region (26).

Our study is the first to analyze comparative 5-year CHE trends and health inequality in western, eastern, and central China, exploring the differences and disparities across regions. We analyze the factors contributing to varying regional CHE trends and the drivers of inter-regional CHE inequality. Using data from three representative eastern-central-western provinces from China Family Panel Studies, we calculated and analyzed the incidence, intensity, and inequality of CHE between 2010 and 2018 to understand inter-regional CHE inequality.

## Methods

### Data

Launched in 2010, with five waves (2010, 2012, 2014, 2016, and 2018) of publicly released datasets, the China Family Panel Studies (CFPS) is a nationally representative longitudinal health survey funded by Peking University (27). No CFPS data are available after 2018, mainly due to the interruption of CFPS by COVID-19. Using implicit stratification and probability-proportional-to-size sampling (PPS), CFPS gathered data on demographics, socioeconomic status, physical and psychological wellbeing, cognitive ability, and healthcare utilization for households with respondents aged 16 years and above.

Covering more than 16,000 households in 25 Chinese provinces, five provinces have adequate provincial-representative samples, comprising three eastern provinces (Shanghai, Guangdong, and Liaoning), Henan in central China, and Gansu in the west. To evaluate regional CHE, we selected Shanghai for the eastern region, Henan for the central region, and Gansu for the western region. As shown in Table 1, Shanghai, Gansu, and Henan are broadly representative of their regions in terms of doctors, medical technicians, and beds per 1,000 residents when compared with the regional averages.

**Table 1:** Population, socioeconomic status, and health resources in the three regions between 2010 and 2018.

**Item**	**Shanghai**		**East Regional average**		**Henan**		**Central Regional average**		**Gansu**		**West Region average**	
	**2010**	**2018**	**2010**	**2018**	**2010**	**2018**	**2010**	**2018**	**2010**	**2018**	**2010**	**2018**
Resident Population (10000 persons)	2303	2424			9405	9605			2560	2637		
Population Density (person/sq.km)	3656	3848			563	575			56	58		
GDP (100 million)	17436.9	32679.9			23157.6	48055.9			4120.8	8246.1		
GDP per capita (RMB)	77275	134982			24516	50152			16113	31336		
Number of physicians per 1,000 people	2.2	3.1	2.5	2.8	1.7	2.5	1.8	2.4	1.5	2.3	1.7	2.4
Number of health technicians per 1,000 people	9.7	8.1	6.2	7.2	3.5	6.5	4.2	6.2	3.7	6.0	4	6.9
Number of beds per 1,000 people	3.7	5.3	4.4	5.6	3.5	6.3	3.5	6.2	3.2	6.2	3.5	6.5

Overall, the 5-wave CFPS included 2,0417 households across the three provinces: 4,448 in 2010, 3,999 in 2012, 4,045 in 2014, 3,964 in 2016, and 3,961 in 2018. After removing missing values and matching household IDs, we constructed an unbalanced panel database with 16,558 households from the 5-wave survey: 4,010 households in Shanghai, 6,128 households in Henan, and 6,420 households in Gansu.

### Variables

A dummy dependent variable (*E*_*i*_) was defined to identify CHE (28) households as household out-of-pocket (OOP) health expenditure, including outpatient and inpatient services, preventative care, maternal and child health services, and medication expenses, equal to or exceeding 40% of the capacity to pay (CTP) for total household necessity expenditure minus food spending. Consistent with previous research (13, 29–31), we collected data on the characteristics of the household and household heads, comprising marital status (married or not married), educational status (illiterate, elementary school, secondary school, and high school and above); type of medical insurance; age (16–35, 36–55, 56–65, and >65); and sex (female and male). Individual self-reported types of medical insurance comprised none (no medical insurance), NRCMS (new rural cooperative medical scheme), URBMI (urban resident basic medical insurance), UEBMI (urban employee basic medical insurance), and other medical insurance (free medical care and supplementary medical insurance). Relevant household characteristics comprised household residence (rural and urban), family size (1–3, 4–6, and >6), whether there was a family member over 60 years old (no and yes), whether a family member had a chronic disease (no and yes), whether a family member received inpatient services in the past year (no and yes), whether a family member received outpatient services in the past 2 weeks (no and yes), and the household's economic status (measured by CPI adjusted per capita income quartile 1 to quartile 4) to compare annual per capita income across different years.

### Measurement of CHE incidence and intensity

CHE incidence was measured by the catastrophic payment headcount (H_cat_) or expenditures as a proportion of household income exceeding the threshold z_cat_, which was 40% of CTP (10). Let *E*_*i*_ be 1 when OOP/CTP≥z_cat_ and 0 otherwise, then H_cat_ is


(1) $$\begin{array}{l} {\text{H}}_{\text{cat}}\;=\;\frac{1}{\text{N}}\overset{\text{N}}{\underset{\text{i }=\;1}{\sum }}{\text{E}}_{\text{i}}\;=\;\mu_{\text{E}},\# \end{array}$$

where N is the sample size and μ_E_ is the mean of E_i_.

In addition, the mean catastrophic gap (G_cat_) was used to measure the intensity of CHE. Let O_i_ be catastrophic overshoot, which equaled OOP/CTP-z_cat_ if E_i_ = 1 and 0 otherwise, G_cat_ is


(2) $$\begin{array}{l} {\text{G}}_{\text{cat}}\;=\;\frac{1}{\text{N}}\overset{\text{N}}{\underset{\text{i }=\;1}{\sum }}{\text{O}}_{\text{i}}\;=\;\mu_{\text{o}}\# \end{array}$$

where N is the sample size, and μ_o_ is the mean of O_i_.

### Measuring inequality in CHE incidence and intensity

**Figure 1:**
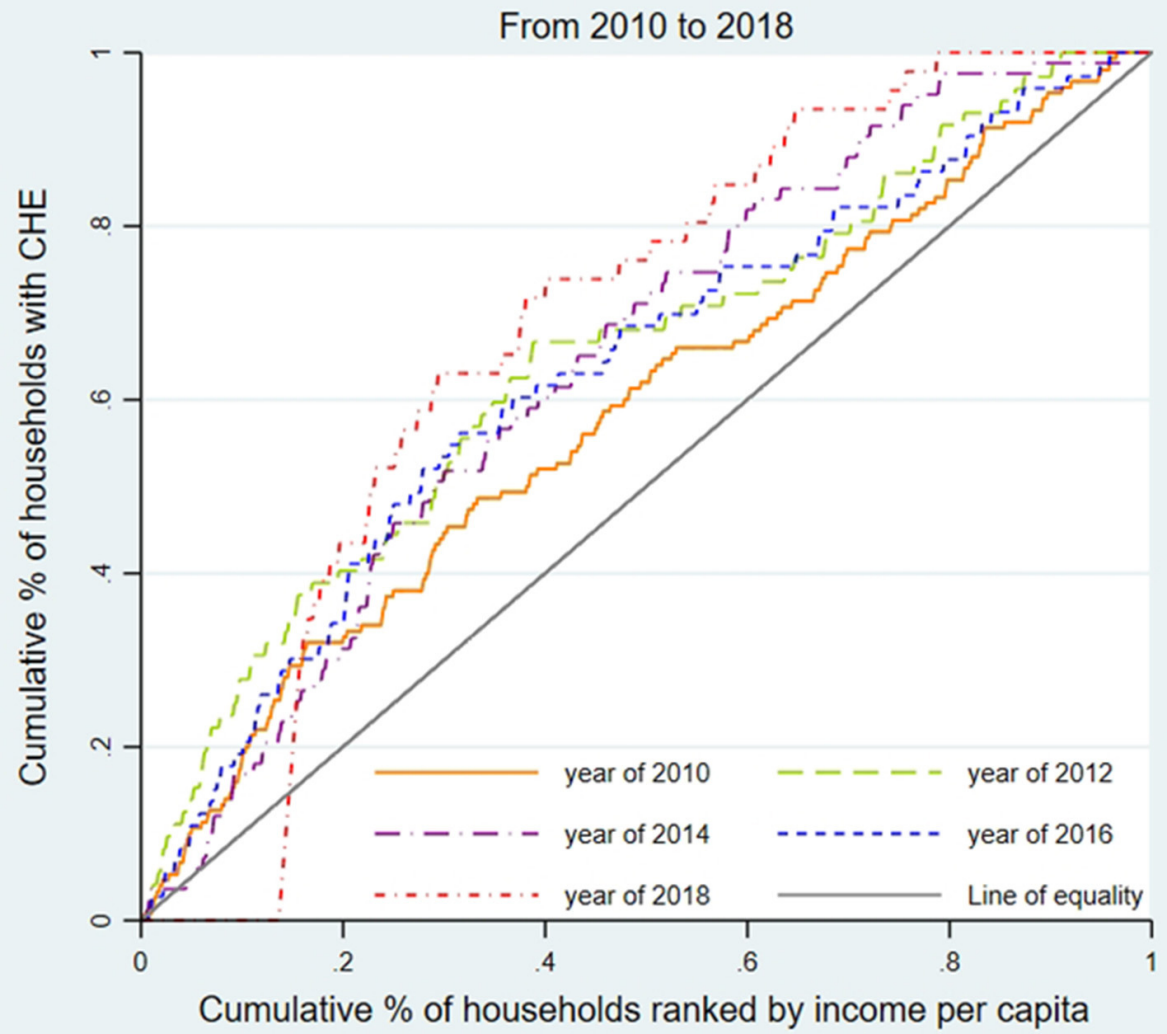
Concentration curve of CHE incidence in Shanghai from 2010 to 2018.

**Figure 2:**
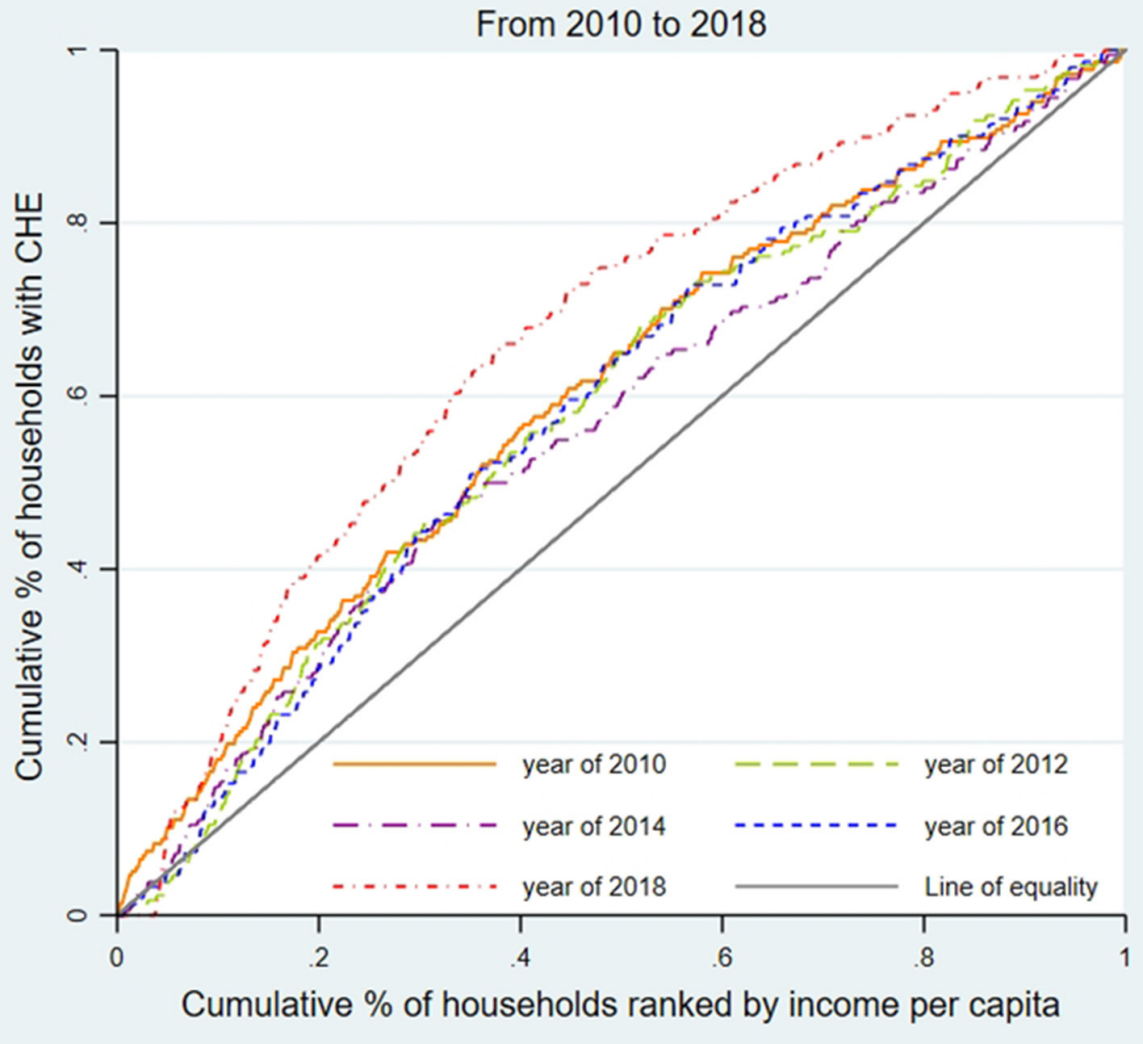
Concentration curve of CHE incidence in Henan from 2010 to 2018.

**Figure 3:**
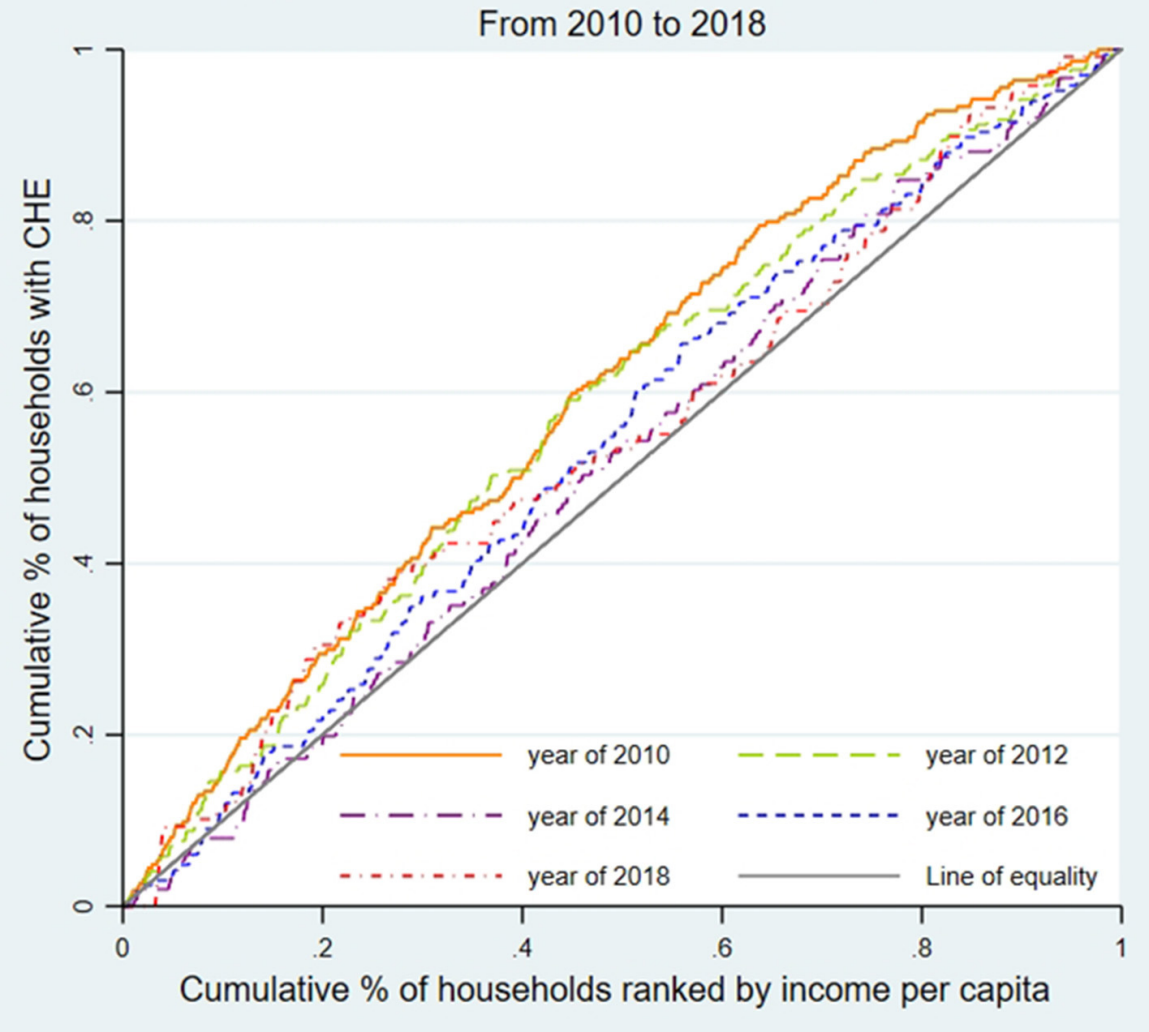
Concentration curve of CHE incidence in Gansu from 2010 to 2018.

We used the concentration curve (CC) and concentration index (CI) to estimate CHE inequality and whether the poor or the better-off were likely to exceed the CHE threshold (31). As shown in Figures 1–3, for each region, we plotted the CC by connecting the cumulative percentage of households ranked by income per capita (x-axis) and the cumulative proportions of households with CHE (y-axis), with the x-axis accumulated from the lowest to the highest. The 45-degree straight line connecting the bottom left to the top right is commonly named 'the line of equality' and represents absolute equality. When the CC is above the line of equality, it indicates a greater tendency for the worse-off to exceed the CHE threshold, while the concentration line below the equality line indicates a greater tendency for the better-off to exceed the CHE threshold.

The CI is defined as twice the area between the CC and the line of equality (32) is as follows:


(3) $$\begin{array}{l} {\text{CI}}_{\text{E}}\;=\;1-2\int_{0}^{1}{\text{L}}_{\text{h}}\text{(p)dp},\# \end{array}$$

where CI_E_ is the CI of CHE incidence and L_h_(p) refers to the CC of CHE incidence. CI_E_ ranges between −1 and 1, where the bigger the absolute value of CI_E_, the worse the inequality of CHE (13). By analogy with the inequality of CHE incidence, we then defined an index (CI_O_) analogous to CI_E_, which captures the CHE intensity inequality.

### Modifying the incidence and intensity of CHE

The standard CHE indices are blind to whether the better or worse-off exceeds the threshold. We used Wagstaff et al.' s (33) W${}_{\text{cat}}^{\text{E}}$ index that weights the variables with the individual's income distribution rank. W${}_{\text{cat}}^{\text{E}}$ is as follows:


(4) $$\begin{array}{l} {\text{W}}_{\text{cat}}^{\text{E}}\;=\;\mu_{\text{E}}\left(1-{\text{CI}}_{\text{E}}\right),\# \end{array}$$

where μ_E_ is the mean of E_i_, and CI_E_ is the CI of CHE incidence. Similarly, we can construct an analog of W${}_{\text{cat}}^{\text{E}}$ to measure modified CHE intensity. Let C_O_ be the CI of the overshoot variable, O_i_, then W${}_{\text{cat}}^{\text{G}}$ could be equal to the following equation:


(5) $$\begin{array}{l} {\text{W}}_{\text{cat}}^{\text{G}}\;=\;\mu_{\text{O}}\left(1-{\text{CI}}_{\text{O}}\right) \end{array}$$

where μ_O_ is the mean of O_i_, and CI_O_ is the CI of CHE intensity. A negative CI_E_ (or CI_O_) indicates a tendency for the excesses to be concentrated among people experiencing poverty, a positive CI_E_ (or CI_O_) indicates that the excesses are mostly concentrated among the rich, and the former underestimates the CHE incidence (or intensity) while the latter overstates it.

### Random effect model

Since the dependent variable was binary, the panel logit model was used to estimate the determinants of CHE occurrence. Considering that the fixed effect model cannot estimate variables that do not vary with time, such as sex, marital status, and educational status, we employed a panel random effect model to analyze the determinants of 2010–2018 CHE occurrence:


(6) $$\begin{array}{l} {\text{Log(P}}_{\text{it}}\text{)}=\text{ln}\left(\frac{{\text{p}}_{\text{it}}}{{\text{1-p}}_{\text{it}}}\right)\text{ = }\alpha_{0}+\alpha_{1}*{\text{household head}}_{\text{i}}\\ \;+\alpha_{2}^{*}{\text{household}}_{\text{i}}+\epsilon_{\text{it}},\# \end{array}$$

where i refers to the *i*_*th*_ household in the three regions, *t* denotes the years 2010, 2012, 2014, 2016, and 2018, $\frac{{\text{p}}_{\text{it}}}{1-{\text{p}}_{\text{it}}}$ refers to the odds ratio of CHE incidence, and ϵ_*it*_ is the random error term.

## Results

### Characteristics of households

Supplementary Table 1 displays the characteristics of households and household heads. From 2010 to 2018, the proportion of household heads over 65 or with a high school education level and above gradually increased, while the proportion of those who were male, married, or without medical insurance decreased. Compared to Henan and Gansu, household heads in Shanghai were more likely to be over 65 years old, women, and better educated (high school and above) but less likely to be married or have medical insurance. Gansu had the lowest proportion of household heads over 65 years old or better educated but the highest proportion of male heads with medical insurance.

According to Supplementary Table 1, between 2010 and 2018, urban households grew rapidly in all three regions. The proportion of urban households in Shanghai was over 80%, which was much higher than that in Henan, which grew from 40% to 50%, and Gansu, which grew from 10% to 30%. Regarding family size, the proportion of households with 1–3 people in Shanghai fell from 75% in 2010 to 69.7% in 2018, in contrast to ~40% of households with 1–3 people in the other two regions. As shown in Supplementary Table 1, from 2010 to 2018, the proportion of household members over 65 years old, suffering from chronic diseases, and receiving inpatient or outpatient services showed an upward trend in all regions. However, Supplementary Table 1 shows significant disparities across the three regions: Shanghai had the highest proportion of household members over 65 years old and with chronic diseases, while Gansu had the lowest. Gansu had the highest percentage of household members receiving inpatient or outpatient services, and Shanghai had the lowest.

### Regional CHE incidence, intensity, and inequality

As shown in Table 2, CHE incidence (*H*_*cat*_) was highest in Henan (except 2016), followed by Gansu and Shanghai. Displaying a similar pattern, CHE intensity (*G*_*cat*_) was highest in Henan (except for 2014), followed by Gansu and then Shanghai. The trend in CHE incidence was downward in all regions, falling from 13.54% to 7.12% in Shanghai, 18.95% to 13.03% in Henan, and 18.53% to 8.48% in Gansu. Intensity also fell across all regions, from 4.56% to 2.67% in Henan province, 4.43% to 1.70% in Gansu, and 3.43% to 1.29% in Shanghai.

**Table 2:** Incidence, intensity, and inequality in the three regions from 2010 to 2018.

**Measures**	**Shanghai**					**Henan**					**Gansu**					**P**
	**2010**	**2012**	**2014**	**2016**	**2018**	**2010**	**2012**	**2014**	**2016**	**2018**	**2010**	**2012**	**2014**	**2016**	**2018**	
**Incidence**																
*H*_*cat*_(%)	13.54	12.59	9.70	8.82	7.12	18.95	16.96	13.37	10.88	13.03	18.53	15.59	11.53	11.76	8.48	< 0.001
$W_{cat}^{E}$(%)	15.86	16.29	12.53	11.15	9.98	22.83	19.91	15.23	12.79	17.47	22.20	18.05	11.99	12.75	9.29	< 0.001
**Intensity**																
*G*_*cat*_(%)	3.43	2.92	2.64	2.17	1.29	4.56	3.70	3.11	2.43	2.67	4.43	3.21	3.74	2.28	1.70	< 0.001
$W_{cat}^{G}$(%)	3.54	3.21	3.15	2.10	1.34	4.79	3.96	3.23	2.49	2.86	4.55	3.24	2.77	2.25	1.82	< 0.001
**Inequality**																
*CI_*E*_*	−0.17	−0.29	−0.29	−0.26	−0.40	−0.21	−0.17	−0.14	−0.18	−0.34	−0.20	−0.16	−0.04	−0.08	−0.10	< 0.001
*CI_*O*_*	−0.03	−0.10	−0.19	0.03	−0.04	−0.05	−0.07	−0.04	−0.02	−0.07	−0.03	−0.01	0.26	0.01	−0.07	< 0.001

The *P*-value is obtained by the Kruskal-Wallis test, and *P* < 0.05 is considered statistically significant.

To assess the inequality of CHE incidence, Figures 1–3 display the concentration curves. The concentration curves were all above the “equity line,” indicating that CHE incidence in all three regions was concentrated among the poor. The figures also show that the trend in inequality changed, first falling and then rising. Shanghai's concentration curve was furthest away from the “fair line,” while Gansu's concentration curve was closest to the “fair line.” For CHE inequality, the concentration index (CI) in Table 2 shows that CI_E_ values in all three regions were negative, with a decreasing, and then rising, trend. The absolute value of CI_E_ was largest in Shanghai (except 2010). We also used CI_O_ to measure the inequality of CHE intensity, which displayed a similar pattern to CI_E_. K-Wallis ANOVA for CI_E_ and CI_O_. Table 2 shows significant differences across the three regions.

In Shanghai, the incidence of CHE (H_cat_) was the lowest among the three regions, while inequality (CI_E_) was the highest. This meant that the poor in Shanghai were more likely to suffer from CHE than in the other two regions.

As for the inequality of CHE intensity, Table 2 shows that CI_O_ values indicate no significant differences across the three regions.

Taking into account the gap between the rich and poor, $W_{cat}^{E}$ shows that CHE incidence has always been lowest in Shanghai and highest in Henan (except in 2014). From the perspective of CHE intensity after the rich-poor adjustment, Henan's CHE intensity was the highest every year, while the CHE intensity of Shanghai was the lowest (except 2014). The K-Wallis analysis of variance showed statistically significant differences in the adjusted incidence and intensity among the three regions.

### Random effect model of CHE incidence

Table 3 shows the results of the random effect model of CHE incidence in the three regions and the odds ratio (OR) corresponding to the 95% confidence interval (CI) and *P*−values.

**Table 3:** Random effect model of CHE incidence in three regions from 2010 to 2018.

**Variables**	**Shanghai** = **4010**		**Henan** = **6128**		**Gansu** = **6420**	
	***OR*(95*%CI*)**	**P**	***OR*(95*%CI*)**	**P**	***OR*(95*%CI*)**	**P**
**Age (reference 16–35)**						
36–55	1.605 (0.814–3.167)	0.172	1.300 (0.883–1.900)	0.185	0.809 (0.608–1.075)	0.143
56–65	2.497 (1.268–4.917)	**0.008**	2.512 (1.679–3.760)	**0.000**	1.193 (0.857–1.662)	0.295
>65	3.933 (1.791–8.638)	**0.001**	4.005 (2.492–6.438)	**0.000**	2.144 (1.419–3.238)	**0.000**
**Gender (reference Female)**						
Male	1.074 (0.805–1.433)	0.628	1.129 (0.917–1.390)	0.253	1.060 (0.857–1.311)	0.592
**Marital status (reference Married)**						
Unmarried or other	1.691 (1.168–2.447)	**0.005**	1.169 (0.863–1.583)	0.312	1.217 (0.917–1.614)	0.173
**Educational status (reference high school and above)**						
Illiterate	1.970 (1.260–3.080)	**0.003**	1.855 (1.314–2.619)	**0.000**	1.713 (1.229–2.389)	**0.002**
Elementary school	1.414 (0.912–2.193)	0.121	1.390 (1.000–1.931)	**0.050**	1.752 (1.243–2.469)	**0.001**
Secondary school	1.099 (0.746–1.621)	0.632	1.048 (0.763–1.441)	0.771	1.165 (0.820–1.657)	0.394
**Medical insurance (reference none)**						
NRCMS	1.192 (0.757–1.876)	0.448	1.007 (0.689–1.471)	0.972	1.021 (0.700–1.490)	0.913
URBMI	1.325 (0.870–2.020)	0.190	1.307 (0.767–2.227)	0.325	1.595 (0.861–2.956)	0.138
UEBMI	0.855 (0.536–1.366)	0.513	0.979 (0.605–1.585)	0.932	0.918 (0.523–1.612)	0.766
Other	1.651 (1.018–2.676)	**0.042**	0.960 (0.530–1.738)	0.892	1.151 (0.586–2.263)	0.683
**Residence (reference urban)**						
Rural	1.213 (0.844–1.743)	0.297	1.230 (0.980–1.544)	0.074	1.510 (1.155–1.974)	**0.003**
**Family size (reference** ≧**6)**						
1–3	5.005 (2.134–11.741)	**0.000**	1.808 (1.391–2.349)	**0.000**	1.640 (1.254–2.144)	**0.000**
4–5	2.227 (0.914–5.426)	0.078	1.366 (1.052–1.775)	**0.019**	1.349 (1.064–1.711)	**0.014**
**Member over 65 (reference No)**						
Yes	1.382 (0.903–2.115)	0.136	1.085 (0.839–1.403)	0.535	0.810 (0.636–1.032)	0.089
**Member with chronic disease (reference no)**						
Yes	1.207 (0.913–1.595)	0.187	1.347 (1.105–1.642)	**0.003**	1.285 (1.066–1.547)	**0.008**
**Member received inpatient services (reference no)**						
Yes	5.680 (4.246–7.600)	**0.000**	4.786 (3.872–5.916)	**0.000**	3.583 (2.935–4.375)	**0.000**
**Member received outpatient services (reference no)**						
Yes	1.545 (1.151–2.073)	**0.004**	1.169 (0.950–1.439)	0.141	1.180 (0.971–1.434)	0.095
**Household's economic status (reference: income quartile 4)**						
Income quartile 1	2.802 (1.806–4.347)	**0.000**	2.937 (2.039–4.231)	**0.000**	2.273 (1.596–3.239)	**0.000**
Income quartile 2	2.169 (1.405–3.350)	**0.000**	2.216 (1.547–3.176)	**0.000**	1.588 (1.113–2.268)	**0.011**
Income quartile 3	1.893 (1.211–2.959)	**0.005**	1.421 (0.985–2.050)	0.060	1.380 (0.963–1.978)	0.079
Constant	0.001 (0.000–0.003)	**0.000**	0.006 (0.003–0.012)	**0.000**	0.012 (0.006–0.024)	**0.000**
lnsig2u	0.044 (−0.554–0.642)	**——**	−0.037 (−0.440–0.366)	**——**	−0.064 (−0.485–0.357)	**——**
sigma_u	1.022 (0.758–1.379)	**——**	0.982 (0.803–1.201)	**——**	0.969 (0.785–1.195)	**——**
Rho	0.241 (0.149–0.366)	**——**	0.227 (0.164–0.305)	**——**	0.222 (0.158–0.303)	**——**
Prob > chi2	**0.000**		**0.000**		**0.000**	
Chi–square	329.91		513.76		362.63	
−2 LL	2117.7392		4129.4882		4433.0958	

^*^Two-tailed *p*-value < 0.05 was statistically significant.

None, households without medical insurance; NRCMS, New Rural Cooperative Medical Scheme; URBMI, Urban Resident Basic Medical Insurance; UEBMI, Urban Employee Basic Medical Insurance; Others, all kinds of relatively high-profit packages of the insurance program. Bold value indicates a significant effect.

The three random effect models were statistically significant after the Wald test, indicating that the non-constant variables of the three models were statistically significant, indicating that the models provided a good fit to the data. For all three regions, households whose heads were over 65 years old (vs. 16–35) or illiterate (vs. high school and above) were more likely to incur CHE, and households with a family size of 1–3 (vs. ≧6), with members who received inpatient services (vs. no), or with the economic status of income quartiles 1 and 2 (vs. income quartile 4) were more likely to incur CHE. Marital status, medical insurance, urban-rural residence, household members with chronic diseases, or receiving outpatient treatment had different effects on the incidence of CHE across the three regions. Sex and households with a member over 65 years old had no significant effect on CHE incidence.

For Shanghai, the incidence of CHE among groups with household heads aged 36–55 years was 2.5 times higher than in the 16–35-year-old group. For households with heads over 65 years, the rate was 3.9 times higher. Compared with those whose household heads had a high school education level and above, the incidence of CHE was two times higher in households with illiterate heads. Those households with members who received inpatient services (vs. no inpatient services, OR = 5.680) or with economic status in income quartile 1 (OR = 2.802), income quartile 2 (OR = 2.169), and income quartile 3 (OR = 1.893) (vs. income quartile 4) were more likely to suffer from CHE. Unlike the western and central regions, marital status, medical insurance, and household members who received outpatient services had significant impacts on CHE incidence in Shanghai: households with unmarried heads were more likely to incur CHE (vs. married, OR = 1.691); household heads with other insurance were more likely to incur CHE (vs. no insurance, OR = 1.651); households with members who received outpatient services tended to incur a higher CHE (vs. no outpatient services, OR = 1.545).

For Henan, households whose heads were aged 56–65 years (OR = 2.512) or over 65 (OR = 4.005) were more likely to suffer CHE than those aged 16–35 years. Household heads who were illiterate were 1.9 times, and those with an elementary school education level were 1.4 times more likely to incur CHE than those with an education level of high school or above. The incidence of CHE in households with a family size of 1–3 was 1.8 times, and in households with a family size of 4–5 was 1.4 times more likely to incur CHE than in households with a family size over 6. Households whose members had chronic diseases (vs. no chronic diseases, OR = 1.347), whose members received inpatient services (vs. no inpatient services, OR = 4.786), or whose economic status was in income quartile 1 (OR = 2.937) and income quartile 2 (OR = 2.216) (vs. quartile 4) had a higher proportion suffering from CHE.

For Gansu households, heads aged over 65 years (vs. age 16–35, OR = 2.144), whose heads were illiterate (OR = 1.713) or with an education level of elementary school (OR = 1.752) (vs. high school and above), were more likely to incur CHE. Households whose family size was 1–3 (OR = 1.640) and 4–5 (OR = 1.349) (vs. family size >6), and whose members had chronic diseases (vs. no chronic diseases, OR = 1.285), whose members received inpatient services (vs. no inpatient services, OR = 3.583), or whose economic status income quartile 1 (OR = 2.273) and income quartile 2 (OR = 1.588) (vs. income quartile 4) were significantly associated with higher CHE incidence. In Gansu, rural households had 1.5 times the probability of incurring CHE than urban households, which was not significant in the other two regions.

## Discussion

This is the first regional study using 5 years of panel data to identify significant variations in CHE incidence, intensity, and equity across China's eastern, central, and western regions.

### Regional CHE incidence and intensity

From 2010 to 2018, CHE incidence fell in all regions. But the absolute level of CHE incidence differed across the three regions: Shanghai had the lowest CHE (2010:13.54%; 2018:7.12%), Gansu always ranked in the middle (2010:18.53%; 2018:8.48%), and Henan had the highest (2010:18.95%; 2018:13.03%). While nationwide CHE studies found the incidence of CHE to be 13% (34, 35), our regional study revealed significant inter-regional differences in CHE incidence.

We are confident that Henan and Shanghai provinces broadly represented their regions. A study of CHE in Shannxi province, a similar central province, found that the CHE incidence dropped from 17.9% to 15.83% in 2008 and 2013, which was comparable to Henan, and a study of eastern Zhejiang province, which is similar in economic structure to Shanghai, had a similar CHE incidence (9.6% in 2015) as Shanghai (14). However, in the West, the fall in Qinghai's CHE incidence (30.5% in 2016) was significantly different from Gansu's, suggesting that further comparative provincial studies should be undertaken (24). Our data indicate that the largest fall in CHE incidence from 2010 to 2018 occurred in the western region (10.05%), while the fall in the eastern region (6.42%) and central region (about 6%) was similar. One reason may be that the economic, social, and health policies targeting the development and poverty relief of the western provinces achieved the aim of significantly lowering CHE in the disadvantaged western region. Investigating the efficacy of different provincial poverty relief programs may also help explain the greater CHE fall in western Gansu and western Qinghai provinces, as poverty relief policies had a differential inter-regional impact.

CHE intensity (G_cat_) reflects the severity of CHE, and its trend is consistent with the incidence of CHE (24). G_cat_ was the lowest in Shanghai (2010: 3.43%, 2018: 1.29%), followed by Gansu (2010: 4.43%, 2018: 1.70%) and Henan (2010: 4.56 %, 2018: 2.67 %), indicating that Shanghai suffered the least severe CHE, followed by Gansu. The CHE mean positive gap (MPG_cat_), the sum of the health expenditure gap of all CHE households and the total number of households with CHE, shows that CHE severely impacted family living standards (36). Shanghai's MPG_cat_ (2010: 25.31%; 2012: 23.19 %; and 2016: 24.66 %) was the highest in most years, while Henan (2010: 24.07%; 2012: 21.82%; and 2018: 22.30%) and Gansu (2010: 23.93 %, 2012: 20.60; and 2018: 19.37 %) were the lowest. Shanghai also had the largest gap between the rich and the poor, and the cost of health services was high, which meant poor Shanghai families were more likely to fall into the 'poverty trap' caused by illness.

### CHE inequality in the three regions

Our CCs and CI_E_ analysis found that people experiencing poverty were more likely to incur CHE in all three regions. CCs in Shanghai deviated the most from the line of equality while Gansu deviated the least, and the CI_E_ of CHE incidence was all negative, with the absolute value of CI_E_ in Shanghai the highest (except in 2010) and Gansu the lowest. Regional inequality problems were worsening over time in all regions. Shanghai experienced the most severe CHE inequality due to the aging of the population, a high incidence of physical disability problems, and increased treatment of chronic diseases, which impacted medical and out-of-pocket health costs (37), exacerbated by limited medical insurance reimbursements or treatments not covered by the insurance reimbursement list. Shanghai's decreased family size constrained the family's ability to share OOP medical costs, increasing the risk of poverty caused by medical expenses. Shanghai's large migrant worker population, substandard working conditions, and poor environmental conditions contributed to the population's unfamiliarity with local medical insurance reimbursement policies, unstable family economic income, and low health status, which helps explain CHE (38). Compared with the other two provinces, Shanghai's CHE inequality reflects the large gap between the rich and the poor. Shanghai's 2018 Gini coefficient, the common indicator of poor-rich income inequality, was 0.458, compared to 0.243 in Henan and 0.342 in Gansu (39). Between 2010 (0.448) and 2018 (0.458), Shanghai's income distribution gap between the rich and poor worsened year by year (39, 40).

### Comparison of influencing factors in CHE incidence

The random effect model revealed that head of household age and education level, household's economic status, family size, and hospitalization affected the incidence of CHE in all regions. CHE was more likely to occur when the head of the household was older due to the older adult having more and more costly diseases and incurring high medical and OOP medical expenditures (41). The higher the education level, the lower the incidence of CHE, possibly because highly educated families had a higher awareness of disease prevention, better healthcare knowledge, and seek timely medical treatment for all attenuating illnesses and their related economic burden (42). The poorer the household's economic status, the higher the incidence of CHE, which is explained by low-income households having fewer financial resources to address large, unplanned medical expenditures (43, 44). CHE was more likely to occur in small families since mutual assistance between family members was weaker than in large families (45, 46). Hospitalization is more likely to lead to CHE since hospitalization imposes extra medical and health costs, many of which are not covered or only partially covered by health insurance (47).

The use of other medical insurance (free medical care and supplementary medical insurance) in eastern China affected the incidence of CHE. Supplementary medical insurance, comprising commercial and enterprise supplementary insurance, was purchased by health-aware patients with a greater risk of illness. Those with supplementary health insurance had large medical expenses, not all covered by their supplementary insurance, incurring OOP medical expenses and causing CHE.

Central and Western region families with chronic diseases were more likely to experience CHE, but not Shanghai families. Table 1 shows that the number of doctors and health technicians in Shanghai was higher than that in Henan and Gansu. Shanghai's family doctor contract service began in 2011, and Shanghai has a stronger primary and hierarchical diagnosis and treatment health services system than the western and central regions, which helps explain the lower CHE for families with chronic disease in Shanghai. Henan and Gansu only implemented the family doctor contract system in 2017, with insufficient doctor numbers, low proficiency of medical technicians, and poor infrastructure, including roads and telecommunications, limiting effective chronic disease monitoring and timely intervention (48).

Patients receiving outpatient services in Shanghai were more likely to experience CHE. This may be related to Shanghai's basic medical insurance policy on outpatient services. Taking 2018 urban and rural resident medical insurance as an example, the deductible for medical insurance reimbursement for outpatient patients aged 19–59 years in Shanghai was RMB500, while for patients over 60 years old, the deductible was RMB300. In Shanghai, the reimbursement ratio was 70% in first-class hospitals, 60% in second-class hospitals, and 50% in third-class hospitals, and outpatient treatment and insurance claims in second-class and third-class hospitals required first-class hospital referrals (49). In contrast, the deductible for Lanzhou, the capital of Gansu Province, was only RMB10 with a 60% reimbursement ratio and an RMB100 reimbursement ceiling per person per year (50). Zhengzhou in Henan province had no deductible for medical insurance outpatient reimbursement, with reimbursement ratios of 45% in municipal medical institutions, 55% in county-level medical institutions, and 65% in village clinics. For compulsory medical insurance, we recommend standardizing deductibles and reimbursement thresholds across provinces and regions to reduce the risk of CHE arising from outpatient services. We also recommend allowing transportability in compulsory household registration medical insurance, especially NRCMS, to support migrant workers' access to urban medical services.

To reduce CHE across regions, we recommend expanding the coverage of China's health insurance coverage, lowering the threshold standard, and providing policies tailored to vulnerable health risk groups, such as older adults and children. Health insurance policies should target the resource-poor population, providing poverty-oriented comprehensive health insurance. We also recommend improving the severe illness medical insurance system to increase the ability of small families to resist the economic risks due to serious and chronic illnesses. To control the excessive growth of health expenses, we recommend that the health insurance prepayment system establish mixed payment methods, such as diagnosis-related groups, to classify types of hospital cases and per capita payments.

The probability of CHE in rural Gansu households was 1.5 times higher than in urban Gansu households, but the urban-rural location was not significant in Shanghai and Henan.

The differences in urban-rural CHE in Gansu may reflect its relatively slow economic development and disparities in per capita disposable income between urban and rural areas. Both health insurance and development policies in western China should target differentials in urban-rural health resources and per capita income levels to address the risk of CHE.

Similar to China's medical insurance framework, other newly industrialized countries, including BRICS countries, developed healthcare policies to mitigate the occurrence of CHE. The Brazilian unified health system (SUS) relies on taxes to fund health insurance, which is protected by the Constitution and covers all country residents (51). Russia also proposed mandatory health insurance, which was mainly financed by government revenue and a social insurance tax. India's universal health insurance, which is 90% funded by government taxes, has greatly reduced out-of-pocket patient costs (52). Whether China's regional experience with CHE and recommendations for reform provide lessons for similar countries will be influenced by country-specific context, healthcare infrastructure and investment differences, and the specific nature of each country's insurance schemes.

### Health poverty alleviation policy and CHE

Provincial Health Poverty Alleviation Policies target poverty, population health, and illness-caused poverty (53). These policies have contributed to the fall in the incidence and intensity of CHE, especially in Gansu and Henan, but have shown mixed results in addressing inequality in the incidence and intensity of CHE. Concentrated among the poor, inequality, the intensity of CHE in the west and central regions first fell and then rose, related to inadequate medical and health resource investment. As shown in Table 1, the number of physicians per thousand population in Gansu (2.25) and Henan (2.45) in 2018 and the number of health technicians per thousand population in Gansu (6.0) and Henan (6.5) were significantly lower than Shanghai physician (3.1) and health technician (8.1) ratios (24). The number of daily visits per doctor was 14.9 in Shanghai, much higher than in Gansu (6.7) and Henan (6.5). Furthermore, in Gansu and Henan, patients sought treatment for ill health from costly city hospitals rather than cheaper local health facilities. The lower average household income and higher percentage of low-income households in Gansu and Henan meant these households faced relatively high out-of-pocket expenses, even when covered by compulsory health insurance (54). We recommend strengthening community and grassroots health facilities to help attenuate health poverty, which requires the integration of health poverty alleviation policy and rural economic revitalization strategies (55).

## Study limitations

We acknowledge several limitations. There were several limitations to the CFPS survey. Only one province in central and western China in the CFPS survey had the required data to assess CHE; CFPS panel data were only available before 2019; there were no CFPS data on the characteristics of hospitals or where adult children support their parents by paying for their medical care, or vice versa; and CFPS provided no data on OOP non-medical direct and indirect costs for medical services, such as transportation expenses, accommodation costs, and income loss, which may lead to an underestimate of the incidence and intensity of CHE. CFPS health service utilization, health expenditure, and household income were self-reported, which may be less accurate than data from medical records. Second, our standard CHE calculation excluded extremely poor households that were unable to afford health services. Future research should focus on identifying families that are too poor to access medical services.

## Conclusion

We revealed significant differences across China's eastern, central, and western regions in CHE incidence, CHE intensity, CHE inequality, and regional differences in the CHE influencing factors. Affected by factors such as the gap between the rich and the poor and the uneven distribution of medical resources, unmarried families in the eastern region, using supplementary medical insurance, and having family members who received outpatient treatment were more likely to experience CHE.

In particular, Shanghai had the largest gap between the rich and the poor, with the living standards of families with CHE in Shanghai severely impacted and likely to be pushed into illness-caused poverty. Families with chronic diseases in the central and western regions were more likely to cause CHE, and rural families in the western region were more likely to experience CHE than urban families. Our findings also highlight the importance of achieving universal health coverage (UHC) to ensure access to high-quality care and services without catastrophic impacts on family budgets. In China, healthcare policy is crucial to achieving this goal. The causes of CHE varied across the different regions, which requires a further tilt of medical resources to the central and western regions, improved prevention and financial support for chronic disease households, the promotion of primary medical care, continued income support for residents in the central and western regions, reform of the insurance reimbursement policy of outpatient medical insurance, and leveling up across regions of healthcare and health insurance policy for vulnerable groups, such as migrants and older adults.

On a regional basis, health policy should not only address CHE incidence and intensity but also its inequality. While China's health poverty alleviation policies have alleviated CHE, especially in poor regions, policy outcomes have a mixed record with the inequality in the incidence and intensity of CHE, which tends to be concentrated among the poor. We recommend that decision-makers adjust and strengthen preventive and healthcare poverty interventions.

## Data availability statement

The original contributions presented in the study are included in the article/Supplementary material, further inquiries can be directed to the corresponding author.

## Author contributions

CL, YG, and XW conducted the literature research, analyzed, and wrote the draft manuscript. YQ conceived and designed the study. SN and EM revised and reviewed the manuscript. All authors read and approved the final manuscript.
