# Supplementary material for: Regional catastrophic health expenditure and health inequality in China
Source: Front Public Health. 2023 Oct 12;11:1193945. doi: 10.3389/fpubh.2023.1193945 (PMC10624124; doi:10.3389/fpubh.2023.1193945)
Supplement: Supplementary file 1 [file Data_Sheet_1.PDF]

Supplementary Table 1 Statistics description of households and household heads in the 3 provinces

Variable	Shanghai					Henan					Gansu				
	2010	2012	2014	2016	2018	2010	2012	2014	2016	2018	2010	2012	2014	2016	2018
**Household Head**															
**Age**															
16-35	122	68	127	128	92	113	94	200	257	213	162	121	179	279	266
	(11.01)	(11.89)	(14.84)	(15.46)	(14.24)	(9.87)	(9.27)	(14.70)	(18.52)	(17.46)	(13.40)	(11.03)	(13.66)	(19.76)	(19.11)
36-55	479	195	290	250	200	626	526	639	615	519	765	677	777	758	736
	(43.23)	(34.09)	(33.88)	(30.19)	(30.96)	(54.67)	(51.87)	(46.95)	(44.31)	(42.54)	(63.28)	(61.71)	(59.31)	(53.68)	(52.87)
56-65	308	177	246	208	148	252	239	288	262	243	206	192	237	234	243
	(27.80)	(30.94)	(28.74)	(25.12)	(22.91)	(22.01)	(23.57)	(21.16)	(18.88)	(19.92)	(17.04)	(17.50)	(18.09)	(16.57)	(17.46)
>65	199	132	193	242	206	154	155	234	254	245	76	107	117	141	147
	(17.96)	(23.08)	(22.55)	(29.23)	(31.89)	(13.45)	(15.29)	(17.19)	(18.30)	(20.08)	(6.29)	(9.75)	(8.93)	(9.99)	(10.56)
**Gender**															
Female	426	177	438	394	324	265	274	648	668	567	207	185	472	495	496
	(38.45)	(30.94)	(51.17)	(47.58)	(50.15)	(23.14)	(27.02)	(47.61)	(48.13)	(46.48)	(17.12)	(16.86)	(36.03)	(35.06)	(35.63)
Male	682	395	418	434	322	880	740	713	720	653	1002	912	838	917	896
	(61.55)	(69.06)	(48.83)	(52.42)	(49.85)	(76.86)	(72.98)	(52.39)	(51.87)	(53.52)	(82.88)	(83.14)	(63.97)	(64.94)	(64.37)
**Marital status**															

Unmarried or other	159 (14.35)	91 (15.91)	140 (16.36)	155 (18.72)	98 (15.17)	110 (9.61)	110 (10.85)	170 (12.49)	176 (12.68)	157 (12.87)	125 (10.34)	109 (9.94)	130 (9.92)	212 (15.01)	210 (15.09)
Married	949 (85.65)	481 (84.09)	716 (83.64)	673 (81.28)	548 (84.83)	1035 (90.39)	904 (89.15)	1191 (87.51)	1212 (87.32)	1063 (87.13)	1084 (89.66)	988 (90.06)	1180 (90.08)	1200 (84.99)	1182 (84.91)
**Educational status**															
Illiterate	191 (17.24)	104 (18.18)	138 (16.12)	138 (16.67)	70 (10.84)	227 (19.83)	244 (24.06)	352 (25.86)	326 (23.49)	234 (19.18)	454 (37.55)	439 (40.02)	555 (42.37)	532 (37.68)	462 (33.19)
Elementary school	178 (16.06)	101 (17.66)	141 (16.47)	139 (16.79)	107 (16.56)	262 (22.88)	253 (24.95)	331 (24.32)	355 (25.58)	265 (21.72)	261 (21.59)	225 (20.51)	261 (19.92)	282 (19.97)	291 (20.91)
Secondary school	359 (32.40)	193 (33.74)	269 (31.43)	250 (30.19)	197 (30.50)	377 (32.93)	290 (28.60)	377 (27.70)	379 (27.31)	391 (32.05)	298 (24.65)	244 (22.24)	270 (20.61)	319 (22.59)	326 (23.42)
High school and above	380 (34.30)	174 (30.42)	308 (35.98)	301 (36.35)	272 (42.11)	279 (24.37)	227 (22.39)	301 (22.12)	328 (23.63)	330 (27.05)	196 (16.21)	189 (17.23)	224 (17.10)	279 (19.76)	313 (22.49)
**Medical insurance**															
None	164 (14.80)	100 (17.48)	82 (9.58)	94 (11.35)	61 (9.44)	109 (9.52)	92 (9.07)	77 (5.66)	74 (5.33)	63 (5.16)	96 (7.94)	46 (4.19)	69 (5.27)	87 (6.16)	59 (4.24)
NRCMS	131 (11.82)	102 (17.83)	141 (16.47)	151 (18.24)	94 (14.55)	727 (63.49)	715 (70.51)	969 (71.20)	1021 (73.56)	859 (70.41)	932 (77.09)	903 (82.32)	1076 (82.14)	1149 (81.37)	1130 (81.18)

URBMI	330	146	254	249	231	54	38	90	78	90	33	26	33	39	63
	(29.78)	(25.52)	(29.67)	(30.07)	(35.76)	(4.72)	(3.75)	(6.61)	(5.62)	(7.38)	(2.73)	(2.37)	(2.52)	(2.76)	(4.53)
UEBMI	197	164	298	280	219	135	127	176	182	187	81	106	110	118	116
	(17.78)	(28.67)	(34.81)	(33.82)	(33.90)	(11.79)	(12.52)	(12.93)	(13.11)	(15.33)	(6.70)	(9.66)	(8.40)	(8.36)	(8.33)
Others	286	60	81	54	41	120	42	49	33	21	67	16	22	19	24
	(25.81)	(10.49)	(9.46)	(6.52)	(6.35)	(10.48)	(4.14)	(3.60)	(2.38)	(1.72)	(5.54)	(1.46)	(1.68)	(1.35)	(1.72)
**Household Residence**															
Rural	189	120	160	105	63	639	574	753	801	659	998	902	1006	1035	964
	(17.09)	(20.98)	(18.69)	(12.68)	(9.75)	(55.81)	(56.61)	(55.33)	(57.71)	(54.02)	(82.55)	(82.22)	(76.79)	(73.30)	(69.25)
Urban	919	452	696	723	583	506	440	608	587	561	211	195	304	377	428
	(82.94)	(79.02)	(81.31)	(87.32)	(90.25)	(44.19)	(43.39)	(44.67)	(42.29)	(45.98)	(17.45)	(17.78)	(23.21)	(26.70)	(30.75)
**Family size**															
1-3	831	419	615	564	450	433	376	532	564	541	353	315	405	496	561
	(75.00)	(73.25)	(71.85)	(68.12)	(69.66)	(37.82)	(37.08)	(39.09)	(40.63)	(44.34)	(29.20)	(28.71)	(30.92)	(35.13)	(40.30)
4-6	237	127	199	220	159	478	364	474	473	402	575	495	558	559	518
	(21.39)	(22.20)	(23.25)	(26.57)	(24.61)	(41.75)	(35.90)	(34.83)	(34.08)	(32.95)	(47.56)	(45.12)	(42.60)	(39.59)	(37.21)
>6	40	26	42	44	37	234	274	355	351	277	281	287	347	357	313
	(3.61)	(4.55)	(4.91)	(5.31)	(5.73)	(20.44)	(27.02)	(26.08)	(25.29)	(22.70)	(23.24)	(26.16)	(26.49)	(25.28)	(22.49)
**Member over 65 years old**															

No	781 (70.49)	374 (65.38)	530 (61.92)	460 (55.56)	327 (50.62)	855 (74.67)	728 (71.79)	921 (67.67)	933 (67.22)	685 (56.15)	950 (78.58)	816 (74.38)	904 (69.01)	968 (68.56)	893 (64.15)
Yes	327 (29.51)	198 (34.62)	326 (38.08)	368 (44.44)	319 (49.38)	290 (25.33)	286 (28.21)	440 (32.33)	455 (32.78)	535 (43.85)	259 (21.42)	281 (25.62)	406 (30.99)	444 (31.44)	499 (35.85)
**Member with chronic disease**															
No	803 (72.47)	363 (63.46)	500 (58.41)	486 (58.70)	350 (54.18)	710 (62.01)	711 (70.12)	873 (64.14)	876 (63.11)	806 (66.07)	758 (62.70)	749 (68.28)	789 (60.23)	843 (59.70)	839 (60.27)
Yes	305 (27.53)	209 (36.54)	356 (41.59)	342 (41.30)	296 (45.82)	435 (37.99)	303 (29.88)	488 (35.86)	512 (36.89)	414 (33.93)	451 (37.30)	348 (31.72)	521 (39.77)	569 (40.30)	553 (39.73)
**Member received inpatient services**															
No	930 (83.94)	457 (79.90)	680 (79.44)	636 (76.81)	497 (76.93)	914 (79.83)	777 (76.63)	990 (72.74)	1017 (73.27)	887 (72.70)	967 (79.98)	814 (74.20)	967 (73.82)	1032 (73.09)	958 (68.82)
Yes	178 (16.06)	115 (20.10)	176 (20.56)	192 (23.19)	149 (23.07)	231 (20.17)	237 (23.37)	371 (27.26)	371 (26.73)	333 (27.30)	242 (20.02)	283 (25.80)	343 (26.18)	380 (26.91)	434 (31.18)
**Member received outpatient services**															
No	959 (86.55)	449 (78.50)	668 (78.04)	647 (78.14)	491 (76.01)	902 (78.78)	775 (76.43)	1011 (74.28)	1051 (75.72)	932 (76.39)	896 (74.11)	822 (74.93)	909 (69.39)	1009 (71.46)	944 (67.82)
Yes	149 (13.45)	123 (21.50)	188 (21.96)	181 (21.86)	155 (23.99)	243 (21.22)	239 (23.57)	350 (25.72)	337 (24.28)	288 (23.61)	313 (25.89)	275 (25.07)	401 (30.61)	403 (28.54)	448 (32.18)

**Household's economic status**															
Quartile 1(lowest)	59<br>(5.32)	39<br>(6.82)	19<br>(2.22)	13<br>(1.57)	162<br>(25.08)	423<br>(36.94)	337<br>(33.23)	307<br>(22.56)	305<br>(21.97)	318<br>(26.07)	613<br>(50.70)	439<br>(40.02)	380<br>(29.01)	366<br>(25.92)	364<br>(26.15)
Quartile 2	152<br>(13.72)	75<br>(13.11)	44<br>(5.14)	23<br>(2.78)	38<br>(5.88)	470<br>(41.05)	315<br>(31.07)	378<br>(27.77)	384<br>(27.67)	274<br>(22.46)	400<br>(33.09)	330<br>(30.08)	424<br>(32.37)	429<br>(30.38)	399<br>(28.66)
Quartile 3	385<br>(34.75)	157<br>(27.45)	188<br>(21.96)	138<br>(16.67)	66<br>(10.22)	193<br>(16.86)	257<br>(25.35)	450<br>(33.06)	439<br>(31.63)	354<br>(29.02)	145<br>(11.99)	247<br>(22.52)	350<br>(26.72)	403<br>(28.54)	368<br>(26.44)
Quartile 4(highest)	512<br>(46.21)	301<br>(52.62)	605<br>(70.68)	654<br>(78.99)	380<br>(58.82)	59<br>(5.15)	105<br>(10.36)	226<br>(16.61)	260<br>(18.73)	274<br>(22.46)	51<br>(4.22)	81<br>(7.38)	156<br>(11.91)	214<br>(15.16)	261<br>(18.75)
